# A rapid diagnostic workflow for cefotaxime-resistant *Escherichia coli* and *Klebsiella pneumoniae* detection from blood cultures by MALDI-TOF mass spectrometry

**DOI:** 10.1371/journal.pone.0185935

**Published:** 2017-10-05

**Authors:** Elena De Carolis, Silvia Paoletti, Domenico Nagel, Antonietta Vella, Enrica Mello, Ivana Palucci, Giulia De Angelis, Tiziana D’Inzeo, Maurizio Sanguinetti, Brunella Posteraro, Teresa Spanu

**Affiliations:** 1 Institute of Microbiology, Università Cattolica del Sacro Cuore, Fondazione Policlinico Universitario Agostino Gemelli, Rome, Italy; 2 Institute of Public Health, Section of Hygiene, Università Cattolica del Sacro Cuore, Fondazione Policlinico Universitario Agostino Gemelli, Rome, Italy; Seconda Universita degli Studi di Napoli, ITALY

## Abstract

**Background:**

Nowadays, the global spread of resistance to oxyimino-cephalosporins in *Enterobacteriaceae* implies the need for novel diagnostics that can rapidly target resistant organisms from these bacterial species.

**Methods:**

In this study, we developed and evaluated a Direct Mass Spectrometry assay for Beta-Lactamase (D-MSBL) that allows direct identification of (oxyimino)cephalosporin-resistant *Escherichia coli* or *Klebsiella pneumoniae* from positive blood cultures (BCs), by using the matrix-assisted laser desorption ionization–time of flight mass spectrometry (MALDI-TOF MS) technology.

**Results:**

The D-MSBL assay was performed on 93 *E*. *coli* or *K*. *pneumoniae* growing BC samples that were shortly co-incubated with cefotaxime (CTX) as the indicator cephalosporin. Susceptibility and resistance defining peaks from the samples’ mass spectra were analyzed by a novel algorithm for bacterial organism classification. The D-MSBL assay allowed discrimination between *E*. *coli* and *K*. *pneumoniae* that were resistant or susceptible to CTX with a sensitivity of 86.8% and a specificity of 98.2%.

**Conclusion:**

The proposed algorithm-based D-MSBL assay, if integrated in the routine laboratory diagnostic workflow, may be useful to enhance the establishment of appropriate antibiotic therapy and to control the threat of oxyimino-cephalosporin resistance in hospital.

## Introduction

In the last decades, bloodstream infections caused by the *Enterobacteriaceae* family-members *Escherichia coli* and *Klebsiella pneumoniae* showing resistance to third-generation oxyimino-cephalosporin antibiotics [1,2], mainly to cefotaxime (CTX) [3], have increased. This has a negative impact on the infection-related mortality rates and hospital costs [4], and it is associated with a delay in the administration of appropriate antibiotic therapy [5]. According to the European Antimicrobial Resistance Surveillance Network data, the percentages of third-generation cephalosporin-resistant *E*. *coli* and *K*. *pneumoniae* isolates ranged from 19.8% and 45.9% in 2011 to 30.1% and 55.9% in 2015, respectively, in Italy alone; conversely, all over Europe the majority of countries reported resistance percentages of 25% or higher [6]. Resistant isolates produce extended-spectrum β-lactamases (ESBLs), the plasmid-mediated enzymes that can confer non-susceptibility to cefotaxime and other oxyimino-cephalosporins (e.g., ceftazidime) and to monobactams [7]. ESBLs are the result of mutations that occur in TEM, SHV, and, more prominently, CTX-M family β-lactamase-encoding genes [3]. CTX-M ESBLs hydrolyze cefotaxime better than ceftazidime, although several CTX-M variants with enhanced ceftazidimase activity have been detected [8]. The worldwide spread of ESBL-producing *Enterobacteriaceae* organisms has led to increased use of carbapenems, resulting in the emergence of plasmid-mediated resistance to carbapenems [9].

In clinical laboratory practice, antimicrobial susceptibility testing is currently performed by using growth-based manual or automated methods, such as agar diffusion assays and broth microdilution systems [10]. However, these phenotypic methods are limited by turnaround times which do not satisfy the demand for timely information about the antimicrobial susceptibility of patient’s isolate. In contrast, molecular genetic methods are timely but able to only provide information about the absence or presence of an ESBL gene, which not always correlates to the phenotype [11]. Reasonably, matrix-assisted laser desorption ionization–time of flight mass spectrometry (MALDI-TOF MS), that is used for successful microbe identification [12], also direct from positive blood cultures (BCs) [13], in many clinical laboratories, has been explored as a tool for rapid antimicrobial resistance detection [14]. In one of these newly generated assays [15], enzymatic cleavage of the β-lactam ring (in the β-lactam antibiotic) leads to specific mass shifts that can be easily monitored by MALDI-TOF MS. In another assay [15], comparison of the growth rates derived from cultivations of the same organism in the presence or absence of an antibiotic (virtually from all classes) enables organism’s susceptibility/resistance status to be assessed.

The aim of the present study was to evaluate the potentiality of a MALDI-TOF MS based assay, reported here as Direct Mass Spectrometry assay for Beta-Lactamase (D-MSBL), for the identification of cefotaxime-resistant *E*. *coli* and *K*. *pneumoniae* directly from positive BCs of patients treated at a tertiary-care Italian hospital.

## Materials and methods

### Study design and samples

The study was approved by the Ethics Committee of our Institution (protocol no. 0022585), but written informed consent was waived since D-MSBL analysis was performed only on a residual portion of clinical sample and patient-identifying information was not included. The study was conducted prospectively between April and December 2015 at the Università Cattolica del Sacro Cuore, Fondazione Policlinico Universitario Agostino Gemelli, which is a large tertiary-care teaching hospital in Rome, Italy. Blood samples were cultured in the BD Bactec FX BC system (Becton Dickinson Instrument Systems, Sparks, MD, USA), using sets of BD Bactec Plus Aerobic and Anaerobic bottles (Becton Dickinson). Gram staining was performed for all positive BCs. If Gram-negative rods were found, BC aliquots were directly used for MALDI-TOF MS identification and D-MSBL analysis (only BC positive for *E*. *coli* or *K*. *pneumoniae* were tested). Conventional cultures of the same sets of BC samples were performed for the isolates’ identification confirmation and reference antimicrobial susceptibility testing. Additionally, contrived samples were spiked at known concentrations of the two *Enterobacteriaceae* species, using bacterial strains with molecularly characterized resistance mechanisms.

### Laboratory methods

#### Direct MALDI-TOF MS analysis

For each sample included in the study, 8 mL of positive BC fluid were inoculated in a VACUETTE Z Serum Sep Clot Activator tube (Greiner Bio-One International GmbH, Frickenhausen, Germany), centrifuged at 3500 rpm for 15 min, and the resulting pellet was transferred in an Eppendorf tube with 1 mL of HPLC-grade water to reach a turbidity of 2 McFarland. After centrifugation at 12000 rpm for 2 min, the supernatant was discarded and the bacterial pellet was used in parallel for direct MALDI-TOF MS identification on a ground-steel target plate with 1 μL of formic acid (≥98%, Sigma-Aldrich, St. Louis, MO, USA) solution and for D-MSBL assay (i.e., β-lactam ring hydrolysis assay) with cefotaxime (CTX, Sigma) (see below). MALDI-TOF MS analyses were performed using a Microflex LT mass spectrometer (Bruker Daltonics, Bremen, Germany). Spectra were analyzed with the Bruker’s Biotyper software (version 3.1), and the Bruker Biotyper library V.4.0.0.1 (5627 entries) was used to achieve bacterial identification [13]. BC samples that were found to contain *E*. *coli* (n = 58) or *K*. *pneumoniae* (n = 35) organisms were subjected to the D-MSBL assay on the same day. To ensure the assay reproducibility, the bacteria (pellets contained 10^8^ CFUs) were resuspended, in triplicate, in 30 μL of CTX solution (0.5 mg/mL). The suspensions were vortexed and incubated at 37°C with agitation (900 rpm) for 1 h. After centrifugation at 13000 rpm for 2 min, 1 μL of supernatant was spotted in duplicate on a polished-steel target plate and covered with 1 μL of HCCA (α-cyano-4-hydroxycinnamic acid) matrix solution (10 mg/mL in 50% acetonitrile). Prior to analysis, the Bruker antibiotic calibration standard, consisting of bradykinin 1–7 (M + H^+^: 757.39 Da), bradykinin 1–5 (M + H^+^: 573.31 Da), Lys-Lys-Lys (M + H^+^: 403.30 Da), and Ser-His (M + H^+^: 243.10 Da), was resuspended in 25 μL of HPLC-grade water and was used for the instrument calibration in the mass range from 100 to 1000 Da. Spectra were acquired automatically in the positive linear mode at a laser frequency of 60 Hz with an acquisition range from 100 to 1000 Da. The peaks corresponding to either non-hydrolyzed or hydrolyzed CTX forms were analyzed using the software ClinProTools (Bruker Daltonics) version 3.0 with the parameters chosen in a mass range between 300 and 600 Da. The reference strains *E*. *coli* ATCC 25922 and *K*. *pneumoniae* ATCC 25955 (β-lactamase non-producers) and *E*. *coli* ATCC 35218 and *K*. *pneumoniae* ATCC 700603 (β-lactamase producers) were used as negative or positive controls, respectively, and were included in every run. For quality control of the CTX solution and for detecting spontaneous hydrolysis, a control without bacteria (CTX only) was also included. All the control samples were processed in the same way as the BC samples. Statistical analysis was performed on the mass peaks corresponding to the spectral profiles acquired from samples consisting of the CTX solution alone, the CTX solution plus test BC fluid, the CTX solution plus positive control (*E*. *coli* or *K*. *pneumoniae* reference strain), and the CTX solution plus negative control (*E*. *coli* or *K*. *pneumoniae* reference strain). Thus, four samples were included in each run of the MALDI-TOF MS analysis. For each sample, a total of 20 mass peaks showing highest intensity were automatically selected, in order to calculate the intensity average (*Ave*) with standard deviation (*StdDev*) of the peaks from all samples by means of the ClinProTools software peak statistic tool. Thus, a test sample was classified as CTX-resistant by an algorithm based on the following equations: *Avex–StdDevx* > *Aves* + *StdDevs*, calculated from CTX-resistance defining mass peaks (370.5 and 414.5 Da), and *Avex* + *StdDevx* < *Aves–StdDevs*, calculated from CTX-susceptibility defining mass peaks (396.5 and 456.5 Da), where “*x*” and “*s*” were the test (unknown) sample and negative (susceptible) sample, respectively; if the equations were not satisfied, the test sample was classified as CTX-susceptible. In some cases, the analysis was conducted on three mass peaks, as the fourth could not be included among the 20 peaks mentioned above (see data in S1 File).

Preliminarily, to validate the D-MSBL assay, we tested 24 bacterial reference strains that were selected to represent the third-generation cephalosporin-resistant strains most commonly isolated in our clinical setting. BD Bactec Plus Aerobic bottles were spiked with 100 μL of a 2-McFarland bacterial suspension obtained with each of 24 ESBL-producing *K*. *pneumoniae* (KPC-3/SHV-11/ TEM-1, n = 4; CTX-M-15/KPC-2, n = 4; CTX-M-15/SHV-28/TEM-1, n = 4) and *E*. *coli* (CTX-M-1, n = 4; CTX-M-15, n = 4; CTX-M-27, n = 4) strains. As above described, BC bottles were incubated in the BD Bactec FX BC system until a positive growth was signaled by the instrument. The BC broths were then processed for the MALDI-TOF MS-based CTX hydrolysis analysis, and the peaks corresponding to non-hydrolyzed or hydrolyzed CTX forms were analyzed, as above described. Thus, each strain was classified according to the above-mentioned algorithm.

#### Conventional culture and susceptibility testing

Aliquots from each positive BC bottle included in the study were subjected to routine Gram stain microscopy, and were subcultured in parallel on a set of selective and nonselective routine agar plates and incubated under appropriate atmospheric conditions for 24 h or re-incubated for 48 h as necessary. Bacterial isolates were identified by the colony-smear method using MALDI-TOF MS, as previously described [13]. For all *E*. *coli* and *K*. *pneumoniae* isolates (n = 93), antimicrobial susceptibility testing (AST) was performed using Vitek 2 AST cards N201 (bioMérieux, Marcy l’Étoile, France). The minimum inhibitory concentration (MIC) results were interpreted according to the EUCAST breakpoints document version 6.0 2016 [10]. The CTX MICs were also determined using the Etest (bioMérieux) to confirm the EUCAST MIC results. Genotypic characterization in all *E*. *coli* and *K*. *pneumoniae* isolates was done by PCR for detection of ESBL or plasmidic AmpC-type enzyme genes (*bla*_CTX-M_, *bla*_NDM_, *bla*_OXA-48_, *bla*_KPC_, *bla*_SHV_, *bla*_TEM_, *bla*_VIM_, *bla*_MOX-1_, *bla*_CMY-2_, *bla*_LAT_, *bla*_DHA-1_, *bla*_ACC_, *bla*_ACT-1_, and *bla*_FOX-1_), using the total DNA extracted from each isolate. PCR amplification was performed with the primers and conditions described by Ye *et al*. [16], and therein references, and the PCR fragments, after purification, were sequenced by a 3100 genetic analyzer instrument (Applied Biosystems, Foster City, CA). Sequences were analyzed by ChromasPro version 2.4.1 (Technelsium Pty Ltd) and database searches were done by BLASTn program of the National Center for Biotechnology Information (NCBI, http://www.ncbi.nlm.nih.gov).

## Results and discussion

All the 24 reference strains of *E*. *coli* and *K*. *pneumoniae* used in the D-MSBL assay validation phase of the study were correctly classified by the MALDI-TOF MS-based algorithm here proposed for detection of CTX resistance. Thus, D-MSBL assay was directly applied on 93 BCs that were detected as positive for *E*. *coli* (58 isolates) and *K*. *pneumoniae* (35 isolates), as documented by the MALDI-TOF MS analysis performed the same day the D-MSBL assay was done, and later confirmed on the isolates grown from subcultures. The D-MSBL assay results were compared with those obtained by the phenotypic or genotypic testing performed on the 93 *E*. *coli* and *K*. *pneumoniae* isolates. The overall results are shown separately for *E*. *coli* and *K*. *pneumoniae*. As it can see for *E*. *coli* (Table 1), 17 of 19 CTX-resistant isolates and 38 of 39 CTX-susceptible isolates were correctly classified as resistant or susceptible, respectively, by the D-MSBL assay.

**Table 1:** Comparison of the cefotaxime resistance mechanism, MIC value, and D-MSBL assay results for 58 *E*. *coli* derived from the BCs tested.

Isolate	Mechanism(s) of resistance	Phenotype		D-MSBL assay
		MIC (mg/L)	Category^a^	Category
1E	CTX-M-1	8	R	R
2E	CTX-M-15	≥64	R	R
3E	CTX-M-15	≥64	R	R
4E	-	≤1	S	S
5E	-	≤1	S	S
6E	-	≤1	S	S
7E	CTX-M-15, SHV-11, TEM-1	≥64	R	S
8E	-	≤1	S	S
9E	-	≤1	S	S
10E	CTX-M-1	≥64	R	S
11E	-	≤1	S	S
12E	CTX-M-27	≥64	R	R
13E	CTX-M-15	≥64	R	R
14E	CTX-M-15	≥64	R	R
15E	CTX-M-27	≥64	R	R
16E	-	≤1	S	R
17E	-	≤1	S	S
18E	CTX-M-15	≥64	R	R
19E	-	≤1	S	S
20E	-	≤1	S	S
21E	CTX-M-15	≥64	R	R
22E	CTX-M-15	≥64	R	R
23E	-	≤1	S	S
24E	-	≤1	S	S
25E	CTX-M-15	≥64	R	R
26E	CTX-M-15	4	R	R
27E	CTX-M-15, TEM-1	≥64	R	R
28E	-	≤1	S	S
29E	-	≤1	S	S
30E	CTX-M-15	≥64	R	R
31E	-	≤1	S	S
32E	-	≤1	S	S
33E	CTX-M-15	≥64	R	R
34E	CTX-M-27	≥64	R	R
35E	CTX-M-15	≥64	R	R
36E	-	≤1	S	S
37E	-	≤1	S	S
38E	-	≤1	S	S
39E	-	≤1	S	S
40E	-	≤1	S	S
41E	-	≤1	S	S
42E	-	≤1	S	S
43E	-	≤1	S	S
44E	-	≤1	S	S
45E	-	≤1	S	S
46E	-	≤1	S	S
47E	-	≤1	S	S
48E	-	≤1	S	S
49E	-	≤1	S	S
50E	-	≤1	S	S
51E	-	≤1	S	S
52E	-	≤1	S	S
53E	-	≤1	S	S
54E	-	≤1	S	S
55E	-	≤1	S	S
56E	-	≤1	S	S
57E	-	≤1	S	S
58E	-	≤1	S	S

^a^The MIC-based category was assessed according to the EUCAST guidelines version 6.0 2016 (S, ≤1 mg/L; R, >2 mg/L).

As it can see for *K*. *pneumoniae* (Table 2), 16 of 19 CTX-resistant isolates and all of 16 CTX-susceptible isolates were correctly classified as resistant or susceptible, respectively, by the D-MSBL assay.

**Table 2:** Comparison of the CTX resistance mechanism, MIC value, and D-MSBL assay results for 35 *K*. *pneumoniae* derived from the BCs tested.

Isolate	Mechanism(s) of resistance	Phenotype		D-MSBL assay
		MIC (μg/mL)	Category^a^	Category
1K	KPC-3, SHV-11, TEM-1	≥64	R	R
2K	-	≤1	S	S
3K	KPC-3, SHV-11, TEM-1	≥64	R	S
4K	KPC-3, SHV-11, TEM-1	≥64	R	R
5K	KPC-3, SHV-11, TEM-1	≥64	R	R
6K	-	≤1	S	S
7K	KPC-3, SHV-12, TEM-1	≥64	R	R
8K	-	≤1	S	S
9K	-	≤1	S	S
10K	-	≤1	S	S
11K	KPC-3, SHV-11, TEM-1	8	R	R
12K	-	≤1	S	S
13K	CTX-M-15, KPC-2	≥64	R	R
14K	KPC-3, SHV-11, TEM-1	≥64	R	R
15K	CTX-M-15, SHV-11	16	R	R
16K	-	≤1	S	S
17K	KPC-3, SHV-11, TEM-1	8	R	R
18K	KPC-3, SHV-11, TEM-1	≥64	R	R
19K	KPC-3, SHV-11, TEM-1	16	R	S
20K	KPC-3, SHV-11, TEM-1	≥64	R	R
21K	KPC-3, SHV-11, TEM-1	≥64	R	R
22K	-	≤1	S	S
23K	-	≤1	S	S
24K	-	≤1	S	S
25K	-	≤1	S	S
26K	CTX-M-15, SHV-38	≥64	R	R
27K	-	≤1	S	S
28K	-	≤1	S	S
29K	-	≤1	S	S
30K	-	≤1	S	S
31K	-	≤1	S	S
32K	CTX-M-15, KPC-3, SHV-11, TEM-1	≥64	R	R
33K	KPC-3, SHV-11, TEM-1	≥64	R	R
34K	CTX-M-15, SHV-28, TEM-1	≥64	R	S
35K	CTX-M-15, KPC-3, SHV-28, TEM-1	≥64	R	R

^a^The MIC-based category was assessed according to the EUCAST guidelines version 6.0 2016 (S, ≤1 mg/L; R, >2 mg/L).

Overall, as shown in Table 3, there were 5 very major errors (2 *E*. *coli* and 3 *K*. *pneumoniae*) and 1 major error (1 *E*. *coli*), that corresponded to those isolates that were incorrectly classified as susceptible (3.4% and 8.6%) or resistant (1.7%), respectively, by the D-MSBL assay.

**Table 3:** Performance of the D-MSBL assay for 93 clinical isolates according to the presence or absence of cefotaxime resistance-associated β-lactamase mutant genes.

	No. of isolates (mutant/wild type)	No. (%) of isolates correctly classified	No. (%) of misclassified isolates	
			VMEs^a^	MEs^b^
Total	93 (38/55)	87/93 (93.5)	5/93 (5.4)	1/93 (1.1)
*E*. *coli*	58 (19/39)	55/58 (94.8)	2/58 (3.4)	1/58 (1.7)
*K*. *pneumoniae*	35 (19/16)	32/35 (92.5)	3/35 (8.6)	-

^a^Very major errors (VMEs) correspond to resistant isolates that were classified as susceptible by the D-MSBL assay.

^b^Major errors (MEs) correspond to susceptible isolates that were classified as resistant by the D-MSBL assay.

**Fig 1:**
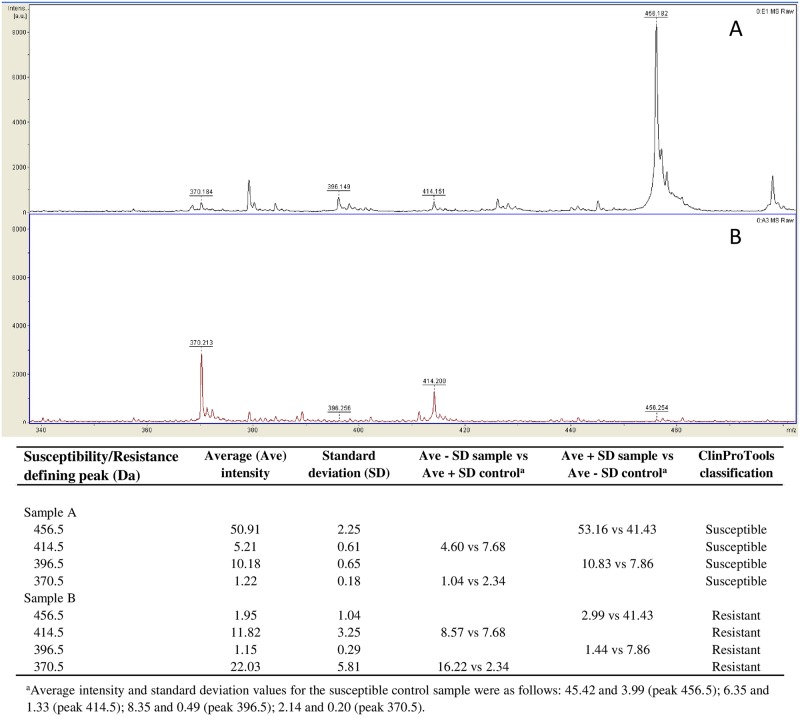
Representative MALDI-TOF mass spectra of two *K*. *pneumoniae* organisms detected as CTX-susceptible (A) and CTX-resistant (B), respectively, by the D-MSBL assay in patients’ BC samples. As detailed on the bottom, classification results were determined by the analysis of both resistance-defining mass peaks (415.5 and 370.5) and susceptibility-defining peaks (456.5 and 396.5) in the test sample compared to those of a control negative (CTX-susceptible) sample (i.e., *K*. *pneumoniae* ATCC 25955 β-lactamase non-producer).

As exemplified in Fig 1, we used a simple algorithm for MALDI-TOF MS analysis to correctly identify CTX-resistant organisms in positive BCs of bacteremic patients, which takes into account the simultaneous reduction of non-hydrolysis peaks (456.5 and 396.5 Da) and increase of hydrolysis peaks (414.5 and 370.5 Da) associated with CTX and its forms [17]. Conversely, the inversion of these peak profiles indicated the presence of a CTX-susceptible organism.

Consistent with previous studies [18,19], our findings correlated well with the CTX resistance levels shown by subcultured organisms via conventional phenotypic testing (MICs from 4 to ≥64 mg/L). Notably, all the CTX-resistant organisms correctly detected by D-MSBL assay were ESBL producers, mainly due to a CTX-M β-lactamase that was possessed by all of 19 *E*. *coli* and 6 of 19 *K*. *pneumoniae* (Tables 1 and 2). The latter organisms were also possessors of a SHV type β-lactamase and/or KPC carbapenemase—specific β-lactamase with the ability to hydrolyze carbapenems [20]. Three *K*. *pneumoniae* isolates with CTX MICs of 16 to ≥64 mg/L were detected as CTX-susceptible organisms by the D-MSBL assay, although these isolates had a resistance mechanism (isolates 3K and 19K, KPC-3/SHV-11/TEM-1; and isolate 34K, CTX-M-15/SHV-28/TEM-1) compatible with the phenotype tested conventionally. Similarly, two *E*. *coli* isolates (MIC, ≥64 mg/L) had a D-MSBL assay result (CTX-susceptible) that was in apparent disagreement with their resistance mechanism (isolate 7E, CTX-M-15/SHV-11/TEM-1; and isolate 10E, CTX-M-1). In one study, Oviaño *et al*. [19] used a second cephalosporin (i.e., ceftazidime) to try to improve the detection of β-lactam resistance in those ESBL cases that were negative with the MALDI-TOF MS assay when CTX was used alone. Thus, it is conceivable that the use of ceftazidime would have enabled detection of our isolates carrying ESBL with a lower rate of CTX hydrolysis and/or higher catalytic efficiency for ceftazidime. However, in 3 of 5 our isolates the ESBL phenotype was totally or partially represented by CTX-M type enzymes.

In summary, we found that the agreement between the D-MSBL assay and the genotypic method results was 94.8% with respect to the *E*. *coli* organisms and 92.5% with respect to the *K*. *pneumoniae* organisms. Data revealed that the assay has an overall sensitivity of 86.8% and specificity of 98.2% in predicting CTX resistance in clinical isolates of *E*. *coli* and *K*. *pneumoniae*. The positive predictive value and negative predictive value were 97.0% and 91.5%, respectively.

**Fig 2:**
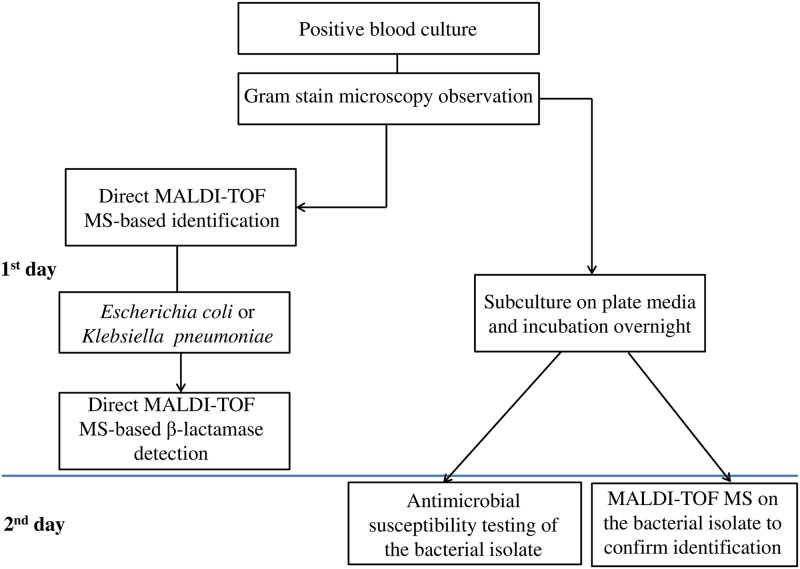
Rapid laboratory flowchart integrating the D-MSBL assay for direct detection of CTX-resistance in *E*. *coli* and *K*. *pneumoniae* from positive blood cultures. The D-MSBL assay results were confirmed by the antimicrobial susceptibility testing that was performed, on the next day, with the bacterial isolates grown from subcultures.

The emergence of multidrug-resistant Gram-negative organisms has a dramatic impact on the patient outcome, and it represents an urgent health-care problem either from a management or an economic standpoint. New and cost-effective diagnostic technologies offer the possibility of overcoming the improper use of antibiotics and, in the meanwhile, controlling the spread of antibiotic-resistant bacteria. Our MALDI-TOF MS-based assay that couples direct identification and detection of CTX-resistant *E*. *coli* and *K*. *pneumoniae* organisms in positive BCs provides rapid results to clinicians who search for the most appropriate antibiotic therapy, without the need for expensive techniques such those based on PCR [11]. The overall expected time is <2 h, taking into account the identification step (30 min) and the D-MSBL assay step (60 min of hydrolysis assay plus 30 min of spectrum acquisition and analysis). Furthermore, our assay has the advantage that a given organism can be classified in the most objective way possible, without the need for presetting species-specific cutoff values to distinguish between susceptible and resistant strains, as recently reported by Jung *et al*. [18]. Therefore, as our study shows, the proposed algorithm-based D-MSBL assay could be integrated into a diagnostic laboratory workflow to detect resistance against cephalosporin antibiotics with a high sensitivity 24 to 48 h earlier than conventional methods (Fig 2).

One limitation of the D-MSBL assay is that it determines susceptibility/resistance to CTX only, thus it does not indicates whether ESBL alone is likely. If this is the case, therapy should be switched to a carbapenem, or if already given, should be continued. Therefore, the assay applies better in epidemiological contexts where the prevalence of infections with carbapenemase-producing bacteria is believed to be low. In our study, none of *E*. *coli* isolates were carbapenem-resistant, in contrast to ~40% of *K*. *pneumoniae* isolates that were found to be resistant to carbapenems. Thus, in our clinical practice, D-MSBL assay may be a valuable tool for early therapeutic guidance in patients with *E*. *coli* bacteremia, whereas only a half of the patients with *K*. *pneumoniae* bacteremia can indeed benefit from this assay. Although this assay holds promise for the future, studies are yet needed to confirm its integration in a laboratory workflow that will allow clinicians to diagnose bacteremia in real time and initiate appropriate antibiotic therapy as early as possible.

## Supporting information

S1 File Supplementary information that includes supplementary tables containing raw data relative to the MALDI-TOF MS analysis performed with the Bruker ClinProTools software. (XLSX)
